# The Effects of Knee Flexion on Tennis Serve Performance of Intermediate Level Tennis Players

**DOI:** 10.3390/s21165254

**Published:** 2021-08-04

**Authors:** Joana Ferreira Hornestam, Thales Rezende Souza, Fabrício Anício Magalhães, Mickäel Begon, Thiago Ribeiro Teles Santos, Sérgio Teixeixa Fonseca

**Affiliations:** 1 Graduate Program in Rehabilitation Sciences, Universidade Federal de Minas Gerais, Belo Horizonte 31270-901, Brazil; hornestam@ufmg.br (J.F.H.); thalesrs@ufmg.br (T.R.S.); fanicio@ufmg.br (F.A.M.); trtsantos@ufmg.br (T.R.T.S.); 2 School of Kinesiology and Exercise Sciences, Faculty of Medicine, Université de Montréal, Montreal, QC H3C 3J7, Canada; mickael.begon@umontreal.ca

**Keywords:** biomechanics, inertial sensors, leg drive, lower limb drive, racket sport, serve speed

## Abstract

This study aimed to investigate the effects of knee flexion during the preparation phase of a serve on the tennis serve performance, using inertial sensors. Thirty-two junior tennis players were divided into two groups based on their maximum knee flexion during the preparation phase of serve: Smaller (SKF) and Greater (GKF) Knee Flexion. Their racket velocity, racket height, and knee extension velocity were compared during the tennis serve. Inertial sensors tracked participants’ shank, thigh, and racket motions while performing five first, flat, and valid serves. Knee flexion was analysed during the preparation phase of serve, knee extension velocity after this phase, racket velocity just before ball impact, and racket height at impact. Pre-impact racket velocity (mean difference [MD] = 3.33 km/h, *p* = 0.004) and the knee extension velocity (MD = 130.30 °/s, *p* = 0.012) were higher in the GKF than SKF; however, racket impact height was not different between groups (*p* = 0.236). This study’s findings support the importance of larger knee flexion during the preparation phase of serve-to-serve performance. This motion should be seen as a contributor to racket velocity.

## 1. Introduction

The serve is one of the most frequent and essential strokes in a tennis match [1]. The serve speed is the most used parameter to evaluate tennis serve performance [2,3,4,5]. A faster serve reduces the time for the opponent to respond and may hamper the return. Another parameter used to measure tennis serve performance is the racket impact height [6,7,8]. Hitting the ball at a higher position may improve the viewing area of the target zone and increase the available target window, increasing the chances to hit faster and more valid serves [2,9]. The upper limb’s angular motion is well known as a major contributor to serve speed [10,11]. However, the influence of the lower limb’s angular motion is still controversial and limited to adult tennis players [5,6,7,12]. More studies are necessary to address the effects of lower limb motion on serve performance, especially in young tennis players.

There is anecdotal evidence that the lower limbs are at the base of the tennis serve’s kinetic chain. Thus, the lower limb motion would be important to initiate energy generation and transfer to the trunk, upper limb, and then to the racket [13]. Greater knee flexion during the serve preparation phase allows reaching greater knee extension velocity (since acceleration is applied during a longer period), producing a more effective lower limb drive as more mechanical energy is added to the body. However, studies that investigated the impact of knee flexion on serve performance found inconsistent results [5,6]. Sgro et al. [6] found that advanced tennis players had greater knee flexion during the preparation phase, greater serve speed, and greater racket impact height than beginners. Moreover, they found a positive association of maximum knee flexion with serve speed and racket impact height. Conversely, Elliott et al. [5] did not find differences in serve speed when comparing tennis players with different lower limb kinematics (greater and smaller knee flexion) during their tennis serve. In addition, studies that induced immediate reduction in knee flexion found it to negatively impact serve performance [7,12]. Artificially restricting maximum knee flexion at 10° with an orthosis [7] or asking players to intentionally reduce knee flexion [12] led to decreases in serve speed and impact height. However, these findings may have been significantly influenced by an unnatural serve motion due to the immediate induction of knee flexion reduction. Therefore, it is still necessary to investigate the influence of the magnitude of knee flexion on tennis serve performance. To the best of our knowledge, no study has compared the serve performance of junior tennis players with different magnitudes of knee flexion in natural serve conditions.

Inertial measurement systems (IMS) are becoming widely used in sports motion analysis [14,15,16,17,18,19,20]. As they enable three-dimensional (3D) motion tracking in sport-specific settings (in-field), they can reveal more realistic results than optoelectronic systems that require laboratory settings. Thus, evaluating the tennis strokes directly on the tennis court using IMS would be interesting to maintain the characteristics of the sport’s natural movement. No previous study has investigated the impact of lower limb motion during serve on tennis serve performance using IMS. Therefore, the current study aimed to investigate on court the effects of the lower limb drive on the tennis serve performance in junior players of intermediate level, using wearable inertial sensors. More specifically, we investigated the effects of the knee flexion magnitude on pre-impact racket velocity and racket–ball impact height during serve in a more ecological condition. Once this effect was confirmed, this study investigated the effect on knee extension velocity as an indicator of lower limb drive effectiveness during the serve. It was hypothesized that junior players of similar levels with greater serve knee flexion would have greater knee extension velocity, higher racket velocity, and higher impact height than those with less knee flexion.

## 2. Materials and Methods

### 2.1. Participants

The sample size was calculated in G*Power software based on a pilot study with 10 participants, considering the differences of the mean pre-impact racket resultant velocity between two groups with different maximum knee flexion during the tennis serve. This calculation indicated a minimum total sample size of 18 participants, considering a power of 90% and alpha of 0.05. Thirty-two junior competitive intermediate-level tennis players, aged between 13 and 17, volunteered to participate in this study. The level of play was defined based on athletes’ International Tennis Number (ITN), which characterizes intermediate players classified as 5 to 7, on a 1–10 scale [21]. By including only intermediate-level tennis players, we limited the tennis level’s influence on serve performance. Indeed, players of different levels could show distinct performances regardless of their knee flexion during the serve [6]. Participants and their legal guardians signed an informed consent form. All players were asymptomatic and none had been injured in the previous six months, had any orthopaedic surgery, or had knee passive range of motion limitations [22]. The participants were equally divided into two groups, with a division cut-off based on the median of the maximum knee flexion (MKF) obtained by the athletes during the serve preparation phase: Greater Knee Flexion group (GKF, *n* = 16) and Smaller Knee Flexion group (SKF, *n* = 16). Due to the unavailability of a clear definition in the literature about high and small values of knee flexion during the serve, this division method was used to guarantee that the two groups had distinct knee flexion values.

### 2.2. Procedures

**Figure 1:**
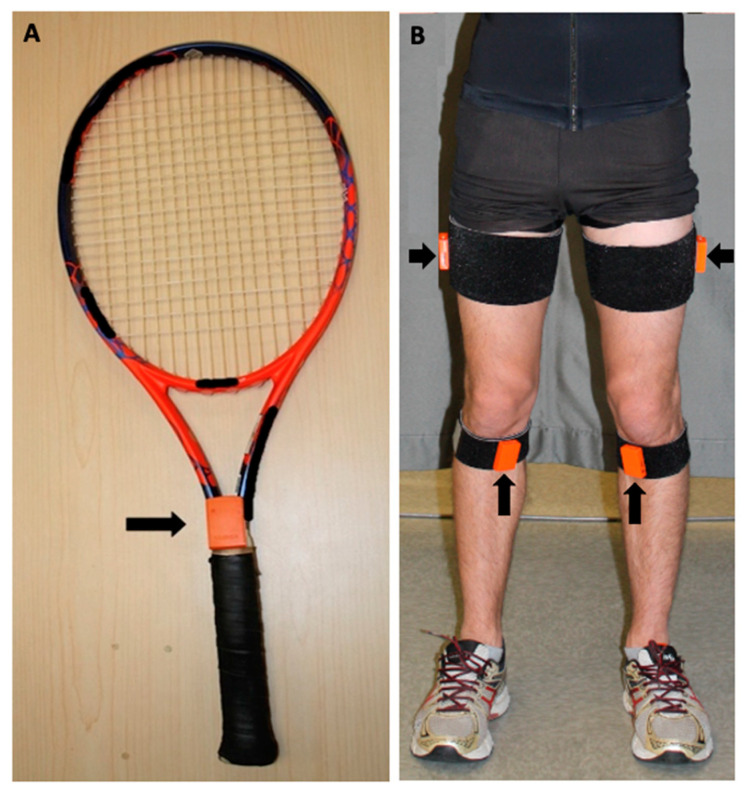
Sensors’ positions. Black arrows indicate sensors location on: (**A**) racket and (**B**) shanks and thighs. Note that motions from only one lower limb (lead leg) were analysed in this study.

An interview to evaluate the volunteer’s eligibility to participate in this study was conducted and followed by body height, weight, and knee passive range of motion (ROM) measurements. These assessments were performed by the same examiner. The participants were asked to warm up as they usually do before tennis practice for 15 min or until they felt they were ready to perform. To test the study’s hypothesis, the racket and knee motions were evaluated. To track them, wireless inertial sensors (called MTw) of the MVN Awinda System (Xsens Technologies B.V., Enschede, The Netherlands) were used. One sensor was placed on the racket right above the grip (Figure 1A), while the other two were placed on the shank and thigh, following the manufacturer’s recommendations [23]. Briefly, to track the knee motion (i.e., shank relative to thigh), one sensor was placed on the tibia’s medial aspect (pes anserinus insertion) and another on the middle of the thigh’s lateral aspect (Figure 1B). Inertial sensors were also placed on other body segments (i.e., foot, pelvis, trunk, upper arm, forearm, and hand) only to allow the system to be calibrated and operate with the full body kinematic model. Data from these sensors were not analysed. Straps with hook-and-loop fasteners were used to keep the sensors in place.

Each inertial sensor (16 g) integrated 3D accelerometers (range ±160 m/s^2^), 3D gyroscopes (range of ±2000 °/s), and 3D magnetometers. The data were sampled at 1000 Hz and the MVN system updated data wirelessly at 60 Hz, which was the maximum permitted by the instrument. The validity of the Xsens MVN System for measuring lower-limb angles has been previously tested in comparison to an optoelectronic motion capture system (MCS), and excellent similarities were found [24,25]. Blair et al. [24] reported insignificant mean differences (range: 0.2–0.3%) for knee flexion during football kicking. Similarly, Al-Amri et al. [25] found excellent similarity between systems for knee flexion-extension during walking, squatting, and jumping (Coefficient of Multiple Correlation (CMC) and R^2^ > 0.9). Likewise, Keaney and Reid [26] examined the validity of a racket inertial sensor for measuring tennis serve speed, compared to an MCS, and found almost perfect agreement between the two systems (ICC = 0.983). To scale the biomechanical model, the ankle, knee, and hip heights in relation to the ground were measured by the same examiner, with the participants in standing position, following the manufacturers’ recommendations [23].

**Figure 2:**
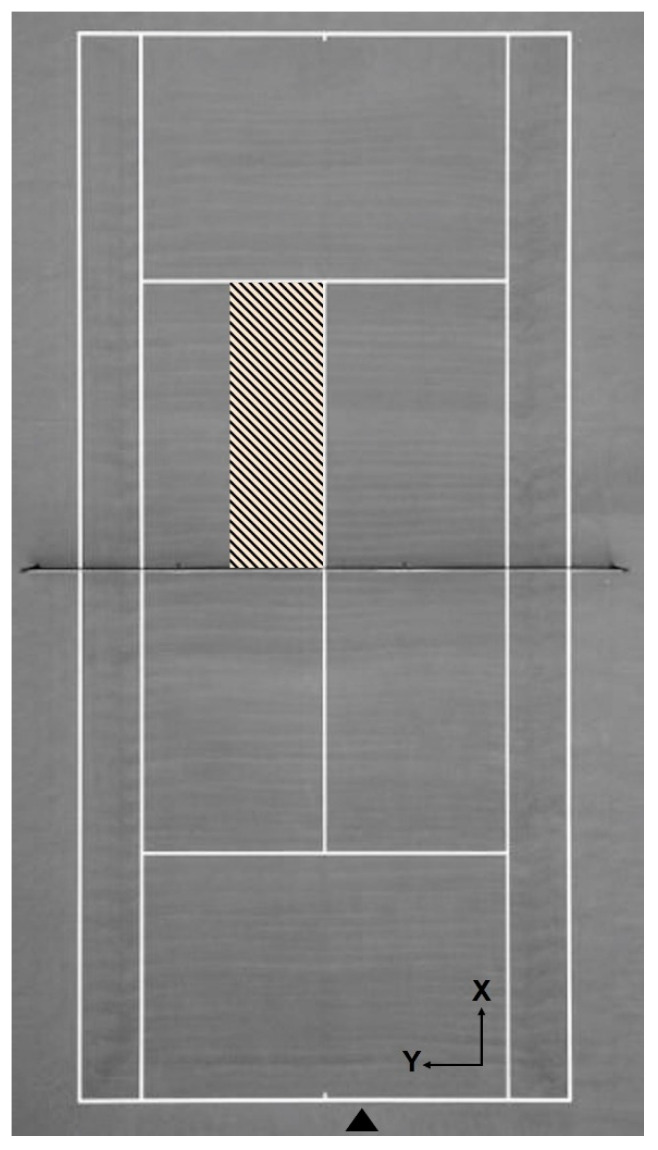
Superior view of the tennis court. The black triangle corresponds to the approximate place where right-hand participants were initially positioned to serve, and the hatched area is the target area. Black arrows indicate X and Y axes in the coordinate systems.

The software MVN Analyze was used to collect and export kinematic data, which were later analysed in the software Visual3D (C-Motion Inc., Germantown, USA) and Matlab (The Mathworks, Natick, MA, USA). The system was calibrated following the N-pose plus walk process, as recommended by the manufacturer [23]. The right-handed participants were asked to serve from the deuce courtside (i.e., the right side of the court) aiming at the target area bordering the “T” (middle) of the service boxes (Figure 2) for 5 min, or until they felt comfortable, to familiarize themselves with the equipment. Left-handed players served from the advantage courtside (i.e., the left side of the court), aiming at the area bordering the “T” of the cross-court service box [27]. The target area was determined using mini disc cones. Finally, the participants were asked to serve only first (i.e., first attempt out of two on a point) and flat serves (i.e., without ball spin) to the target area, and five valid serves were considered for analysis. One observer was positioned near the target area to confirm serves were valid (ball landing in the target area). Players used their rackets, which had similar size and weight characteristics, to guarantee they were used to the equipment while serving. Players’ serves were videotaped with a video camera (Xsens Technologies B.V., Enschede, The Netherlands) positioned on the baseline using a tripod and operating at 60 fps (maximum frame rate possible). These images were only used to confirm the racket–ball impact moment.

### 2.3. Data Processing and Reduction

Global and local coordinate systems (X, Y, Z) were determined in the software MVN Analyze, taking into account the tennis court orientation: *X*-axis was defined as anterior–posterior, *Y*-axis as medial–lateral, and *Z*-axis as vertical (Figure 2). The MVNX files, which contain the biomechanical model and the motion files, were exported from this software and imported into Visual3D software for analysis. Using the built-in functions of Visual3D pipelines, the front (i.e., lead leg) knee flexion angle was calculated based on the shank’s position and orientation relative to the thigh around *Y*-axis, using the Y-X-Z Cardan sequence, i.e., flexion, abduction, rotation [28]. Similarly, the front knee extension velocity was also calculated. The front knee corresponded to the left knee of the right-handed players and the right knee of the left-handed players.

The serve preparation phase was determined on the Visual3D from the racket’s maximum anterior position to the maximum knee flexion [27]. Racket–ball impact was identified as being the highest racket linear velocity in the anterior direction [29]. The maximum knee extension velocity was calculated between the serve events MKF and racket–ball impact. These two serve events were also visually inspected and confirmed in Visual3D and in the recorded videos. Pre-impact racket resultant velocity was calculated in the Matlab as the norm of racket’s linear velocity in the three planes of motion, just before (1 frame) the racket–ball impact [11,30,31]. Racket impact height (i.e., the racket’s vertical distance to the ground) was also obtained and expressed as a percentage of the standing height of the participant (normalized racket height). The mean of five valid tennis serve trials from each participant was used for analysis.

### 2.4. Reliability

A pilot study with ten subjects was conducted to investigate the study’s measures and procedure reliability. Data were collected in two different sessions, one week apart. The test–retest reliability (ICC_2,5_) for maximum knee flexion during the preparation phase of serve, maximum knee extension velocity, pre-impact racket resultant velocity, and racket impact height were 0.93, 0.94, 0.89, and 0.88, respectively.

### 2.5. Statistical Analysis

Descriptive statistics were used for participants’ characterization. Normality was tested using Shapiro–Wilk’s test. A chi-squared test was used to compare groups for ITN and sex. Independent samples *t*-tests were used to compare groups for the maximum knee extension velocity, pre-impact racket velocity, and normalized racket height at impact. The mean differences (MD) between groups and Cohen’s *d* effect sizes (ES) [32] were calculated. All analyses were performed in SPSS software (IBM Corp., Armonk, NY, USA) with a significance level of α = 0.05.

## 3. Results

### 3.1. Participants

There were thirteen right-handed and three left-handed, and fourteen right-handed and two left-handed participants in the SKF and GKF groups, respectively. No difference between SKF and GKF groups was found for the descriptive variables, except for the serve maximum knee flexion that was used to divide groups and was found to be 19.08° greater in the GKF (Table 1). Results of the chi-squared test also showed no difference between groups for the level of play (ITN) (*p* = 0.494) and sex (*p* = 0.194).

### 3.2. Tennis Serve Performance and Knee Extension Velocity

**Figure 3:**
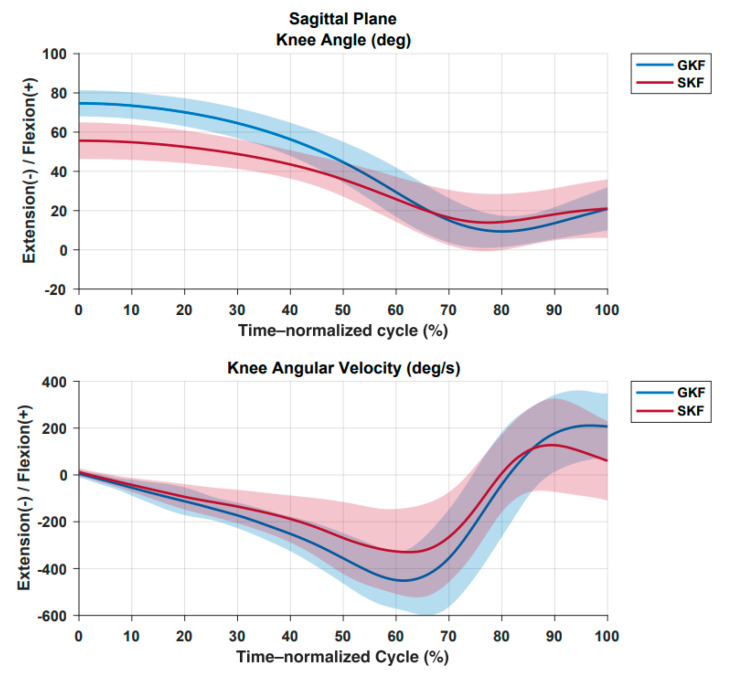
Knee motion in the sagittal plane. Top: knee angle. Bottom: knee angular velocity. Solid lines: mean values. Shadows: standard deviation. Blue: greater knee flexion group (GKF). Red: smaller knee flexion group (SKF). The serve cycle was time–normalized between the events “maximum knee flexion” and “racket–ball impact”.

The pre-impact racket velocity was 3.33 km/h higher and the maximum knee extension velocity was 130.30 °/s higher in GKF than SKF. There was no statistical difference between groups for the normalized racket impact height (Table 2). Time series for the descriptive data of knee angle and angular velocity in the sagittal plane are presented in Figure 3.

## 4. Discussion

This study aimed to investigate the influence of the knee flexion magnitude during the serve preparation phase on knee extension velocity and the tennis serve performance of junior competitive players. To improve the ecological validity of the results, this study was performed on a tennis court using wireless inertial sensors. We found that tennis players with greater knee flexion during the preparation phase of their serve had 32% higher knee extension velocity and 16% higher pre-impact racket velocity than players with less knee flexion. The greater knee angular velocity may have contributed to the greater racket velocity found in this study. Nevertheless, the normalized racket impact height was not significantly different between groups. These findings suggest that tennis coaches and players should consider the magnitude of knee flexion when planning training to improve serve performance.

The greater pre-impact racket velocity found in participants with greater knee flexion may be explained by increases in knee extension velocity and possibly in mechanical energy generation and transfer throughout the kinetic chain. Increased knee flexion during the preparation phase of a serve typically leads to increases in the range of knee extension during the lower limb drive phase (propulsion) of the serve [12]. Displacement throughout a greater joint range of motion seems to be related to greater joint velocity. As found in the present study, Anderson and Sidaway [33], who analysed the soccer kick, also found that players who flexed their knee more during the preparation phase had greater maximum knee extension velocity during the acceleration phase. This association between knee flexion angle and extension velocity may be explained by the fact that covering a greater joint range of motion would give the individuals more time to apply acceleration and increase joint velocity. Complementarily, another possible explanation relies on the stretch-shortening cycle (SSC) function. As the knee flexes during the serve preparation phase, the quadriceps contract eccentrically [8], which may result in elastic energy storage [34,35]. This energy may be used, at least partially, to increase knee extension velocity during serve. Due to the relationship between joint velocity and kinetic energy, it is expected that increased knee extension velocity during a serve increases the energy generated by lower limbs, which is typically transferred, through the kinetic chain, to the trunk, upper limb, and finally to the racket. This mechanism would ultimately increase the serve speed [36,37]. The pre-impact racket velocity is strongly correlated with the post-impact ball speed [10]. Thus, pre-impact racket velocity is commonly reported as an indicator of serve performance [7,30,31].

Our results corroborate with Sgro et al. [6], who also found that players with greater knee flexion were also the ones with faster serves. However, they divided groups based on the participants’ level of play and the group with greater knee flexion and faster serve were advanced tennis players, who were compared with lower level players. Therefore, their results were influenced by the player’s game level, while participants in our study were all at the same level (intermediate) with no statistical difference between groups. Additionally, in the current study, no differences were found between groups for other variables that may influence serve performance—such as body height and mass, age, tennis experience time, and weekly training volume—addressed in previous studies [3,38,39,40].

Contrary to our results, Elliott et al. [5] found no difference in professional players’ serve speed regardless of their knee flexion efficiency. However, they reported the angle of knee flexion at maximum shoulder external rotation (i.e., after the preparation phase), when the knee is typically already extending [36]. While greater knee flexion at maximum shoulder external rotation could be due to a greater knee flexion during the preparation phase [5], it is also reasonable to believe that the opposite could be true. Speculatively, players with less knee flexion at maximum shoulder external rotation could be the ones who flexed their knee more during the preparation phase of a serve and had a more effective lower limb drive. Therefore, examining knee flexion during the preparation phase is a more appropriate method to evaluate the contribution of knee flexion to serve performance.

In contrast to our hypothesis, the normalized racket impact height was not different between players with different knee flexion magnitudes. Although the players with greater knee flexion had a more effective lower limb drive and, therefore, a potential to reach higher, their racket impact height was not greater, as we expected. Our result agrees with Girard et al. [8], who did not find an association between vertical ground reaction force and racket impact height during the serve of intermediate-level tennis players. Although these authors did not measure knee flexion, the vertical ground reaction force is expected to increase as knee flexion increases during the serve preparation phase [7]. A possible explanation for these findings relies on a dependency of racket impact height on other factors, such as the ball toss height and shoulder mobility, which were not measured. As the racket height at impact depends on the ball location, if the ball toss height is low, for example, the player will not hit at higher locations. Similarly, shoulder mobility deficits may limit the player’s ability to hit the ball higher at serve impact.

The maximum knee flexion values found in the present study, using an inertial measurement system, were within the range found in other studies that used optoelectronic motion analysis [12,27] or videography systems [6]. However, the pre-impact racket resultant velocity was lower than the reported in other studies [30,31]. This difference may be explained by the differences in the sample’s level of play and methods used. The participants of Gillet et al. [30] and Rogowski et al.’s [31] studies were advanced tennis players (ITN 2 to 4), whereas the participants of our study were intermediate level. It is known that the level of play impacts serve speed. Higher play levels are related to greater serve speed [6,39]. The difference in racket velocity may also be explained by the different methods for measuring the racket’s velocity. Gillet et al. [30] and Rogowski et al. [31] reported the velocity at the centre of the racket face. In contrast, in the current study, the inertial sensor to track racket velocity was placed on the top of the grip (Figure 1). Mitchell et al. [29] found differences of up to 70% when comparing racket velocity measured at the centre of the racket face and around the middle portion of the grip. Our results seem to agree with the literature if considering the location on the racket where the velocity was tracked.

The method used in the present study brings some limitations that should be discussed. The inertial system’s wireless update rate may be considered low (60 Hz), which was the instrument’s maximum frequency. However, each inertial sensor internally sampled data at a high frequency (1000 Hz), which helped maintain acquisition accuracy during dynamic motion. Another limitation was the fact that this study did not investigate the effects of greater knee flexion during the preparation phase of the serve on upper joint motions (trunk and dominant upper limb). Since these joint motions could also be related to serve performance, more studies must explore these effects. Moreover, the neuromusculoskeletal maturity (e.g., growth rate) of the studied adolescents, which affects their physical capabilities and coordination, was not controlled. However, all participants were competitive athletes with approximately seven years of tennis experience, and so their high skill level may have helped to overcome the possible impact of physiological changes (not measured) on their performance.

To the best of our knowledge, this is the first study to investigate the effects of lower limb motion on serve performance on the tennis court (sport-specific setting) with an inertial measuring system. This approach indicates that using these types of sensors can provide more realistic analyses than those performed in a laboratory setting. Sports scientists and professionals can rely on the method used and consider the results obtained in their practice. However, further studies are necessary to investigate the effects of specific methods to increase knee flexion during the preparation phase on serve performance.

## 5. Conclusions

The serve pre-impact racket velocity of junior tennis players of intermediate level with greater knee flexion during the serve preparation phase was higher than for those with less knee flexion. Additionally, greater knee extension velocity was found in this group, indicating a more effective lower limb drive during the serve. However, the racket impact height was not different between groups. The magnitude of knee flexion should be seen as a contributor to the pre-impact racket velocity.

## Figures and Tables

**Table 1:** Descriptive data of the participants.

Descriptive Data	SKF (*n* = 16)	GKF (*n* = 16)	*p*-Value
Body height (m) ^a^	1.66 ± 0.08	1.67 ± 0.06	0.942
Body mass (kg) ^a^	54.75 ± 6.25	56.08 ± 6.69	0.567
Age (years) ^b^	13.81 ± 1.05	14.25 ± 1.24	0.305
Tennis playing experience (years) ^a^	6.50 ± 2.42	7.00 ± 2.10	0.537
Weekly tennis training (h) ^b^	8.75 ± 1.44	9.25 ± 1.24	0.361
Weekly conditioning training (h) ^b^	4.38 ± 0.72	4.63 ± 0.62	0.361
Serve maximum knee flexion (°) ^a^	55.64 ± 8.66	74.72 ± 5.88	<0.001 *

Results are reported as mean and standard deviation. * *p* < 0.001. SKF: Smaller Knee Flexion group. GKF: Greater Knee Flexion group. ^a^ Variables normally distributed. *p*-values from the independent samples *t*-test are reported above. ^b^ Variables not normally distributed. *p*-values from the Mann–Whitney test are reported above.

**Table 2:** Comparative table of the tennis serve performance and knee extension velocity.

Tennis Serve Performance	Descriptive	SKF (*n* = 16)	GKF (*n* = 16)	*p*	ES
Racket resultant velocity (km/h)	Mean ± SD	21.12 ± 3.76	24.45 ± 1.73	0.004 *	1.138 (large)
	CI_95%_	19.12 − 23.13	23.52 − 25.37		
Normalized racket impact height (%)	Mean ± SD	122.63 ± 5.30	124.49 ± 3.16	0.236	0.426
	CI_95%_	119.80 − 125.45	122.81 − 126.17		
Maximum knee extension velocity (°/s)	Mean ± SD	405.11 ± 160.45	535.41 ± 110.74	0.012 *	0.945 (large)
	CI_95%_	319.61 − 490.61	476.40 − 594.42		

SKF: Smaller Knee Flexion group. GKF: Greater Knee Flexion group. *p*: *p*-values. ES: Cohen’s *d* effect size. SD: standard deviation. CI_95%_: 95% confidence interval. * *p* < 0.05.
